# Non-structural protein 1 and hematology parameters as predictors of dengue virus infection severity in Indonesia

**DOI:** 10.25122/jml-2022-0300

**Published:** 2023-10

**Authors:** I Gusti Agung Ayu Eka Putri Sunari, Aryati Aryati, Faradila Khoirun Nisa Hakim, May Fanny Tanzilia, Nelly Zuroidah, Billy Jordan Wrahatnala, Ali Rohman, Puspa Wardhani, Dominicus Husada, Muhammad Miftahussurur

**Affiliations:** 1 Department of Clinical Pathology, Faculty of Medicine, Dr. Soetomo Teaching Hospital, Universitas Airlangga, Surabaya, Indonesia; 2 Faculty of Medicine, Universitas Airlangga, Surabaya, Indonesia; 3 Department of Chemistry, Faculty of Science and Technology, Universitas Airlangga, Surabaya, Indonesia; 4 Department of Child Health, Faculty of Medicine, Dr. Soetomo Teaching Hospital, Universitas Airlangga, Surabaya, Indonesia; 5 Department of Internal Medicine, Division of Gastroentero-Hepatology, Faculty of Medicine, Dr. Soetomo Teaching Hospital, Universitas Airlangga, Surabaya, Indonesia; 6 *Helicobacter pylori* and Microbiota Study Group Institute of Tropical Disease, Universitas Airlangga, Surabaya, Indonesia

**Keywords:** NS1, dengue virus, severity, infectious disease, human and disease

## Abstract

Dengue virus infection (DVI) remains a significant health challenge, and diagnosis must still be considered. Non-structural protein 1 (NS1) is a potential marker of the dengue virus that can help diagnose DVI. The study aimed to assess the role of NS1 as a predictor of the severity of DVI. We utilized Dengue PCR-confirmed samples and employed semi-quantitative NS1Ag ELISA for NS1 examination, adhering to the World Health Organization South-East Asia Region (WHO-SEARO) 2011 criteria for DVI. We included DVI patients from Indonesia aged 1-65 years. Secondary infections had more severe clinical conditions than primary infections. Leukocyte and platelet levels had a more significant effect on NS1 positivity (6.19 (1.9-30.2); p<0.001; 190 (11-417); p=0.015; respectively). Multivariate analysis revealed leukocytes as a more significant predictor of NS1 values than platelets, with an odds ratio of 5.38 contributing to 30.5% of the NS1 value variation. The NS1 value could distinguish undifferentiated fever and dengue fever in the children group with a sensitivity of 76.0% and specificity of 87.5% (p=0.015). The number of NS1(-) in the severe dengue hemorrhagic fever (DHF) group was higher than NS1(+). DENV-4 type and primary infection were dominant in this study, although they did not significantly differ from the NS1 value. NS1 value can be used as a predictor to determine the severity of DVI in children but not in the adult group. The levels of leukocytes and platelets influenced the NS1 value.

## INTRODUCTION

Dengue virus infection (DVI) is an infectious disease caused by the Flavivirus family Flaviviridae and spread by the *Aedesaegypti* mosquito. Dengue virus is a single-stranded ribonucleic acid (RNA) virus with four serotypes, namely DENV-1, DENV-2, DENV-3, and DENV-4. These four serotypes have similar antigenic structures but are genetically different. The differences in nucleotide sequences between serotypes and genotypes cause variations in biological properties and antigenicity [1]. The pathogenesis of DVI has yet to be fully explained. Various hypotheses have emerged to explain the pathogenesis of DVI, including antibody-dependent enhancement (ADE), T-cell mediated, viral virulence, and molecular mimicry. The most accepted hypothesis in explaining the development of dengue hemorrhagic fever and dengue shock syndrome is the ADE theory [2].

The incidence of DVI worldwide has increased 30 times in 50 years. DVI often gives a clinical picture that is not typical and can resemble flu symptoms, typhoid fever, and several other disease symptoms [3]. WHO-SEARO 2011 divides the clinical manifestations of DVI into asymptomatic and symptomatic. Clinical symptoms are divided into undifferentiated fever, dengue fever without or with signs of bleeding, dengue hemorrhagic fever without or with shock, and expanded dengue syndrome. The most common signs and symptoms are fever, headache, retro-orbital pain, myalgia, and hemorrhagic manifestations. Leukocytes, platelets, and increased hematocrit are key laboratory parameters used to differentiate the severity of DVI [4].

East Java Province ranks among the regions in Indonesia most affected by dengue, reporting approximately 7,838 cases. Jember and Tulungagung regencies within this province have demonstrated particularly high case fatality rates (CFR) for dengue hemorrhagic fever (DHF) in 2017, recorded at 1.5% and 3.1%, respectively [5]. Data from the East Java Provincial Health Office (2019) further showed that Tulungagung regency had 870 dengue cases from January to September 2019, with a marked increase in incidence during the early months of the year, specifically January, February, and March [6].

NS1 is a non-structural protein of the dengue virus with a molecular weight of 46-50 kDa. This protein is produced by dengue virus-infected cells in the form of secreted nonstructural protein 1 (sNS1) [7, 8]. Secreted NS1, rich in lipids associated with glycosaminoglycans (GAGs), facilitates NS1 attachment to cell membranes. The similarity of the structure of NS1 with high-density lipids (HDL) is thought to be the cause of NS1, which can interfere with the coagulation cascade through interaction interference or biogenesis [9]. During the acute phase of infection, circulating levels of sNS1 can be detected in the serum of patients [10], with reported concentrations ranging from 0.01 to 50 µg/mL [1]. These circulating levels of NS1 vary depending on factors such as the serotype of the dengue virus, the immune status of the host (primary or secondary infections), and the severity of the disease [11].

The selection of diagnostic methods is essential to establish an accurate diagnosis. The NS1 antigen examination has a higher confidence level than the antibody examination [12]. Detection and quantification of circulating free NS1 antigen can be a specific diagnosis of DVI and can predict disease severity earlier than Immunoglobulin M (IgM) serological tests [13]. Previous studies found that the sensitivity value of NS1 enzyme-linked immunosorbent assay (ELISA) in primary viral infections was higher than in secondary infections [14, 15]. A previous study mentioned that an increase in sNS1 levels to 600 ng/mL within 72 hours could indicate a heightened risk of progression to DHF [13]. Another study found that NS1Ag levels exceeding >48.49 panbio units were associated with a risk of causing shock in patients with DVI [16]. Slightly different from other studies, NS1 levels could not be used as a predictor of the severity of DVI [17].

In addition to the severity influenced by NS1 antigen, several studies also mention the relationship between NS1 antigen levels and the status of dengue infection. Another study noted that secondary infections had more severe clinical manifestations than primary infections, so they needed to be differentiated [18]. Other studies revealed differences in NS1 levels between primary and secondary infection [19]. This difference in results prompted researchers to evaluate whether the NS1 value could be used to predict the severity of DVI.

## MATERIAL AND METHODS

### Research methods and samples

This research is an analytic observational study employing a cross-sectional design. All samples were tested with DENV Reverse Transcription Polymerase Chain Reaction (RT-PCR), with positive results subsequently confirmed through Dengue NS1 ELISA testing. The study population included patients aged 1-65 with complaints of fever for less than five days who came for treatment or hospitalization at hospitals and health centers in Jember and Tulungagung, Indonesia, from March 2019 to February 2020. One hundred ninety-nine serum samples were collected and stored in a freezer at -70 °C. Out of 199 samples, 66 were positive for PCR examination and were selected for this study. We assumed that samples with positive DENV RT-PCR were confirmed DVI patients. Dengue RT-PCR examination was conducted at the Dengue Laboratory, Eijkman Institute for Molecular Biology, Jakarta. A total of 60 samples in this study were also used in Aryati *et al*. research on the molecular epidemiology of the dengue virus [10].

### NS1 antigen detection kit

The detection of NS1 antigen was carried out using the Platelia Dengue NS1 AG kit from Bio-Rad (France). This kit is a one-step enzyme-linked immunoassay for qualitative or semi-qualitative detection of dengue virus NS1 antigen. The principle of the kit examination in detecting NS1Ag is the sandwich enzyme microplate immunoassay with murine monoclonal Ab peroxidase as the probe. The sample works according to the manufacturer’s instructions. The examination results are in the form of a S/CO ratio, where S is the optical density of the sample, and CO is the average calibration repetition. The ratio values were interpreted as follows: a S/CO ratio greater than one indicated a positive result, a S/CO ratio less than 0.50 signified a negative result, and a S/CO ratio between 0.50 and one was considered equivocal.

### DENV serotype PCR detection kit

PCR detection of the DENV serotype uses multiplex real-time reverse transcription polymerase chain reaction (RT-PCR) from abTES™ DEN/CHIKU 5qPCR kit (AIT Biotech, Singapore). This reagent can simultaneously detect DENV-1, DENV-2, DENV-3, DENV-4, and CHIKV (Chikungunya virus) in one reaction tube, with a total amount of 140 µL of a serum sample for examination according to the instrument’s instructions.

### Dengue IgM and IgG kit to determine the type of infection

The examination of dengue IgM and IgG was conducted using the Panbio Dengue IgG/IgM Capture Enzyme-Linked Immunosorbent assay (ELISA) kit (Panbio Diagnostic, Brisbane, Australia). The principle of the examination is indirect ELISA. Sample work was carried out according to the manufacturer's instructions. The results were categorized into positive, negative, and equivocal. A primary infection was indicated if the IgM level exceeded 11 Panbio units, the IgG level exceeded 22 Panbiounits, and the IgM/IgG ratio was above 1.2. Conversely, a secondary infection was suggested if the IgM level was less than 11 Panbio units, the IgG level was above 22 Panbio units, and the IgM/IgG ratio was below 1.2.

### DVI severity according to WHO-SEARO 2011

In this study, the World Health Organization South-East Asia Region Office (WHO-SEARO) 2011 criteria were employed to categorize the severity of DVI. The WHO-SEARO 2011 guidelines define several categories of DVI, including undifferentiated fever (UF), dengue fever (DF), dengue hemorrhagic fever (grades I, II, III, IV), and expanded dengue syndrome.

The classification of DVI severity is based on a comprehensive assessment that includes physical examination findings, laboratory results, and other dengue-specific markers, if available. Key clinical symptoms evaluated in this process include fever, headache, retro-orbital pain, myalgia, arthralgia, rash, and signs of plasma leakage. Laboratory parameters considered were the presence or absence of leukopenia, thrombocytopenia, and an increase in hematocrit [2].

### Data analysis

The Kruskall-Wallis comparison test was used to determine the relationship between NS1 values and the severity of DVI WHO-SEARO 2011. The relationship between NS1 values and infection status was analyzed using the Mann-Whitney test. The Receiver Operating Characteristic (ROC) test was used to determine the semi-quantitative NS1Ag value. Statistical analysis was carried out using SPSS 17.

## RESULTS

### Sample characteristics

Based on the interpretation of NS1, 66 samples were divided into 29 NS1 positives, 36 NS1 negatives, and one NS1 equivocal. Equivocal results were excluded from the analysis because they were doubtful. The demographic distribution of the samples showed an equal number of male participants across both NS1+ and NS1- groups. However, there were more female participants in the NS1- group than in the NS1+ group. Additionally, the NS1- group had a higher proportion of adult participants than children. Changes in hematocrit were not included because not all patients could be evaluated for changes in hematocrit. The sensitivity of NS1 to PCR was found to be 44%. The characteristics of the research sample can be seen in Table 1. Significant differences (p-value<0.05) were observed only in leukocyte and platelet counts.

**Table 1:** Sample characteristics based on NS1 interpretation

Parameter	NS1(+)	NS1 (-)	p-value
**Male/Female**	17/12	17/19	0.364^a^
**Child/adult (mean±2SD)**	16/13(19.45±13.68)	13/23(19.74±5.33)	0.127^a^
**Leukocytes (%)** <4x10^3^/µL 4-10x10^3^/µL >10x10^3^/µL	19 (28.8) 10 (15.2) -	9 (13.6) 19 (28.8) 8 (12.1)	<0.001^b^
**Platelets (%)** <20x10^3^/µL 20-50x10^3^/µL 50-100x10^3^/µL >100x10^3^µL	1 (1.5) 3 (4.5) 12 (18.2) 13 (19.7)	1 (1.5) 3 (4.5) 4 (6,1) 28 (42.4)	0.015^b^
**Serotype (%)** DENV-1 DENV-2 DENV-3 DENV-4	4 (6.1) 2 (3) 6 (10.6) 16 (24.2)	1 (1.5) 1 (1.5) 6 (9.1) 28 (42.4)	0.067^b^
**Primary/Secondary Infections (%)**	21/8 (31.8/12.1)	31/5 (47/7.6)	0.091^a^
**WHO-SEARO-2011 DVI Severity (%)** Undifferentiated fever Dengue Fever DHF grade 1 DHF grade II DHF grade III DHF grade IV (DSS)	2 (3) 18 (27.3) 6 (9.1) - 3 (4.5) -	12 (18.2) 13 (19.7) 6 (9.1) 1 (1.5) 4 (6,1) -	0.111^b^

a Value of p with Mann Whitney test. ^b^ Value of p with Kruskal-Wallis test. DHF: dengue hemorrhagic fever; DSS: dengue shock syndrome.

Table 1 shows that leukocyte and platelet counts were significantly associated with NS1 values. Two other parameters that approached statistical significance were the dengue virus serotype (p=0.067) and the type of infection (p=0.019), while other parameters did not show significant associations. A multivariate analysis was conducted to determine the parameters strongly affecting the NS1 value, adjusting for age and sex. Leukocyte count significantly impacted NS1 values (p=0.004), with a regression coefficient of 1.683 and an odds ratio (OR) of 5.38. This suggests that a lower leukocyte count was associated with a fivefold increase in the likelihood of a positive NS1 result. Likewise, platelet count (p=0.019) obtained a regression coefficient of 1.388 and an OR of 4.01. The lower the level of platelets, the higher the probability of a positive NS1 result. The level of leukocytes had a more substantial influence on the NS1 value than platelet count. Leukocytes and platelets affected the NS1 value by 30.5% (R^2^ 0.305).

The probability of a positive NS1 value was influenced by the levels of leukocytes and platelets in the blood. Specifically, when leukocyte levels were below 4x10^3/L, there was a 55% chance of a positive NS1 test result. Similarly, if platelet counts dropped below 100x10^3/L, the likelihood of a positive NS1 result increased to 47.5%. If both levels were combined, the percentage increased to 82.9%.

### NS1 value to differentiate severity

The NS1 value was obtained based on the value of the S/CO ratio. We analyzed all positive DENV PCR samples to determine the association with severity. The normality test results indicated a non-normal data distribution, as evidenced by a p-value of less than 0.05. Spearman’s analysis of the NS1 value with the 2011 WHO-SEARO severity criteria was not significant (p-value= 0.111). However, when the data was stratified by age and severity based on WHO-SEARO 2011, a significant difference was observed in children (p=0.015), while no significant correlation was found in adults (p=0.937) (Table 2).

**Table 2:** Distribution and value of NS1 stratified by age and WHO-SEARO 2011 severity

WHO-SEARO 2011 Dengue Criteria	Age group			
	Children		Adults	
	n	p-value	n	p-value
**Severity (%)** Undifferentiated fever Dengue Fever DHF grade 1 DHF grade II DHF grade III DHF grade IV (DSS)	30 (45.5) 8 (12.1) 13 (19.7) 6 (9.1) 1 (1.5) 2 (3) -	0.015^a^	36 (54.5) 7 (10.6) 18 (27.3) 6 (9.1) - 5 (7.6) -	0.937^a^

DHF: dengue hemorrhagic fever; DSS: dengue shock syndrome. ^a^ p-value, Kruskal-Wallis test.

**Figure 1:**
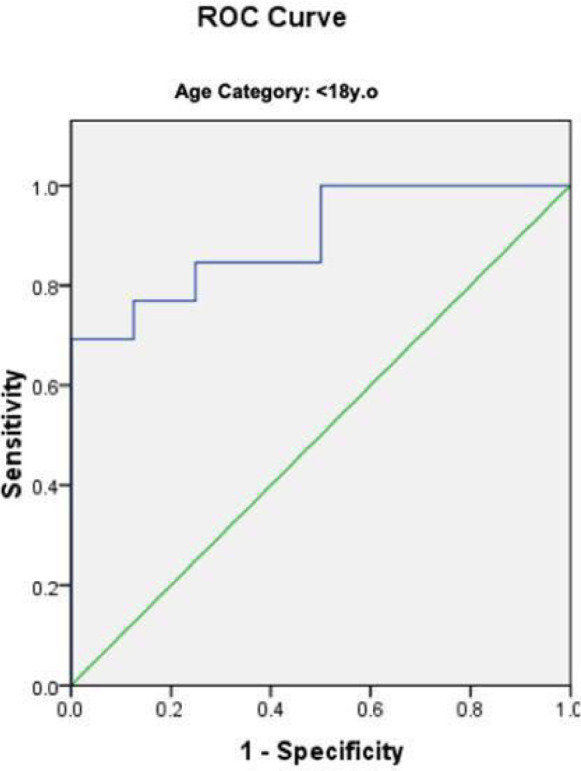
ROC curve comparing undifferentiated fever and dengue fever among children

ROC analysis was carried out in the children group to determine the NS1 cutoff value for the severity of DVI according to WHO-SEARO 2011. A total of 30 samples were divided based on the WHO-SEARO 2011 DVI criteria, namely eight samples with undifferentiated fever, 13 with dengue fever, 6 with DHF I, one with DHF II, and two with DHF grade III. Best sensitivity and specificity values were obtained by comparing the UF and DF groups. The sensitivity value was 77.9%, the specificity was 87.5%, and the area under the curve (AUC) was 0.894 (Figure 1). The cutoff value of the NS1 ELISA ratio was 0.276. The determined cutoff value for the NS1 ELISA ratio was 0.276, indicating a negative interpretation. This finding suggests a negative NS1 value does not necessarily rule out dengue virus infection.

### Relationship between NS1Ag value and infection status

Overall, there were more patients with primary infection than secondary infection. The proportion of primary infections was higher in the NS1(-) group, accounting for 58.5%, compared to 39.6% in the NS1(+) group. The Mann-Whitney test, applied to evaluate if the NS1 values could differentiate between primary and secondary infections, had a p-value of 0.091, indicating no statistically significant difference between the NS1Ag ratio values and the infection status. Consequently, it can be said that the NS1 value could not distinguish between primary and secondary infections.

## DISCUSSION

Determining the NS1 ELISA cutoff value as a predictor of severity has yet to be widely carried out. NS1 can be used as a predictor, considering that NS1 can damage the endothelium directly and become a toll-receptor four agonist that stimulates myeloid cells to produce cytokines [20]. Several studies have found changes in NS1 value in more severe conditions, but no one has researched NS1 in states of mild infection [13, 16]. In this study, NS1 could differentiate between undifferentiated and dengue fever in children but not in the adult group. We confirm that this is the first study to find an NS1 cutoff value in mild DVI in the pediatric cohort. Researchers believe that NS1 also affects the adult group [16]. Further research is needed to evaluate other factors not covered in this study.

Hematology parameters that affected NS1 in this study were platelets and leukocytes. Another study reported that a single NS1Ag examination correlated with thrombocytopenia, and this correlation was even stronger when combined with antibody tests [21]. Leukopenia is often found in patients with DVI due to direct suppression of the bone marrow [22]. We identified seven patients with thrombocyte counts below 100,000 µL, supporting previous studies where thrombocytopenia was observed in patients with negative NS1 [23]. Multivariate analysis showed that leukocytes and platelets predicted a positive NS1 result. Lower levels of these parameters were associated with a higher likelihood of positive NS1 values, contributing to 30.5% of the NS1 value variance. The remaining variance was likely due to other factors not examined in this study. Previous studies stated that platelet activation is influenced by DENV NS1, which causes aggregation, attachment to endothelial cells, and phagocytosis by immune cells, resulting in thrombocytopenia [24]. Similar to previous studies, the value of leukocytes, especially lymphocytes, had a significant relationship with the value of NS1 [16]. These results highlight the strong correlation between the levels of platelets and leukocytes and NS1 values and serve as potential predictors of DVI severity alongside NS1.

NS1 value is highly valuable for the diagnosis of DVI. The NS1 ELISA method has a higher confidence than the dengue antibody test [12]. However, the sensitivity of NS1 in detecting DVI can vary significantly, as evidenced by the low sensitivity observed in this study. This variation aligns with findings from other research, which also report diverse sensitivity levels for NS1 [25-27]. Several factors contribute to the variability in NS1 sensitivity, including the serotype and genotype of the dengue virus, viral load, host immune response, infection status, and the type of diagnostic reagent used [28]. The sensitivity of the NS1 antigen in this study was notably lower compared to a previous study that utilized the same diagnostic kit [29]. This discrepancy in sensitivity levels may be attributed to the different serotypes of the DENV predominant in each study. In this research, the DENV-4 serotype was more prevalent, whereas the previous study primarily encountered the DENV-1 serotype, which explains why the sensitivity was lower in this study. The effect of the DENV-4 serotype on NS1Ag sensitivity has been reported, with the sensitivity of NS1 being low, almost the same as the sensitivity of this study [30]. Patients with DENV-4 infection were reported to be more susceptible to cutaneous complaints such as maculopapular rash and erythema, as well as respiratory complaints such as pharyngeal congestion [31].

This study detected that the number of patients with NS1(-) results was higher than those with NS1(+) results. It was observed that patients with a negative NS1 result could still progress to severe dengue. Another study stated that a single examination of NS1 using the ELISA method had a sensitivity of 67% and a specificity of 99%. This suggests that a negative NS1 result does not conclusively rule out dengue infection [32]. The decrease in NS1 value in severe clinical conditions can be caused by soluble NS1, which interacts with complement proteins and forms a soluble complement-fixing (SCF) antigen [9]. In addition, low NS1 value can be caused by a large number of NS1 trapped in the immune complex, and monoclonal antibodies can bind to a limited amount of NS1, resulting in low or even negative readings [28].

The type of infection also influences clinical manifestation severity. Secondary infection is more severe than primary infection due to antibody-dependent enhancement (ADE) [33]. NS1Ag sensitivity in secondary infection was lower than in primary infection [34]. In this study, primary infection was more common than secondary infection, and mild severity was more predominant than severe conditions in patients with DENV-4 infection. This is similar to another study that stated that primary DENV-4 infection had milder clinical manifestations than secondary DENV-2 infection [25]. In this study, the NS1 value was not related to the infection status; thus, the cutoff value could not be determined. This is distinct from a study that found a range of NS1Ag levels in primary and secondary infection [19].

## CONCLUSION

The NS1 value can be used as a predictor to measure the severity of DVI, but it is necessary to pay attention to the type of infection, virus serotype, and other parameters in determining the severity of DVI.

## Data Availability

The data of ROC analysis to determine the cutoff value of NS1Ag can be accessed at the following link: https://doi.org/10.6084/m9.figshare.20387970.
